# Laser-Ablated Tungsten as an Interfacial Architecture for Tantalum Coatings: Suppression of Shutdown-Stage Water Corrosion in Accelerator-Driven Neutron-Source Targets

**DOI:** 10.3390/nano16161043

**Published:** 2026-08-21

**Authors:** Baolong Ma, Shixi Chen, Yaru Wang, Fanxi Zhang, Sheng Wang, Yupeng Xie

**Affiliations:** 1 School of Nuclear Science and Technology, School of Energy and Power Engineering, Xi’an Jiaotong University, Xi’an 710049, China; baolongma@xjtu.edu.cn (B.M.);; 2 State Industry-Education Integration Center for Medical Innovations, Xi’an Jiaotong University, Xi’an 710049, China; 3 XJTU-Huzhou Neutron Science Laboratory, Science Valley Medium-Sized Building #1, Huzhou 313000, China; 4 HBNCT Co., Ltd., Hangzhou 310000, China; 5 National Key Laboratory for High Energy Pulsed Power, Xi’an Jiaotong University, Xi’an 710049, China

**Keywords:** tungsten target, tantalum coating, laser ablation, static deionized-water corrosion, surface engineering, neutron source

## Abstract

Water-cooled tungsten targets may remain in contact with stagnant deionized water during accelerator shutdown, creating a beam-off corrosion condition distinct from irradiation-assisted service. Untreated tungsten (W), laser-ablated tungsten (LA-W), and a 500 nm tantalum-coated laser-ablated tungsten surface (Ta-LA-W) were therefore immersed in deionized water for up to 28 days. Laser ablation replaced the machined surface with a hierarchical micro/nanostructure and increased the areal roughness Sa from 0.22 ± 0.01 to 1.75 ± 0.07 μm; after Ta deposition, Sa was 1.62 μm. LA-W exhibited the largest topographic attenuation and the highest dissolved W concentration, reaching 7.915 mg·L^−1^ at 21 days. Ta-LA-W maintained Sa within the range from 1.60 ± 0.06 to 1.66 ± 0.07 μm and limited dissolved W to 1.118–1.601 mg·L^−1^. X-ray photoelectron spectroscopy showed increased WOx-related intensity and disappearance of the metallic-W loss feature only for untreated W. The W 4f envelopes of LA-W and Ta-LA-W remained broadly similar before and after immersion, but for different reasons: sustained W dissolution for LA-W and suppression of W oxidation and release by the Ta-containing barrier for Ta-LA-W. The dissolution-based corrosion sequence was LA-W > W > Ta-LA-W. Laser texturing therefore acted as an interfacial-engineering treatment rather than an intrinsically corrosion-resistant modification.

## 1. Introduction

Accelerator-driven neutron sources encompass large proton-spallation facilities and compact systems based on proton, deuteron, or electron accelerators. In each configuration, the neutron-production target must convert concentrated beam power into a useful neutron field while preserving dimensional stability, heat-transfer capability, and compatibility with the coolant and surrounding structural materials [1,2]. Tungsten is attractive for these applications because its high atomic number and density support efficient spallation or bremsstrahlung–photonuclear neutron production, while its high melting point provides a wide thermal margin. Tungsten-based target–moderator–reflector assemblies have consequently been developed for both high-power and compact neutron-source concepts [3,4,5].

A substantial fraction of the incident beam energy is deposited as heat in or near the target, and water cooling is commonly used to remove this thermal load. During beam operation, coolant flow, elevated temperature, radiolytic oxidants, irradiation-induced defects, and cyclic stress can act simultaneously at the target–coolant interface. During scheduled or unscheduled shutdown, however, the beam and forced circulation may be interrupted while deionized water remains in contact with the target. Corrosion then proceeds under a stagnant or weakly refreshed beam-off condition. Although this exposure does not reproduce the coupled irradiation, thermal, and hydrodynamic environment of operation, repeated shutdown periods may affect coolant contamination, surface-product accumulation, restart cleanliness, and long-term interface integrity.

The aqueous corrosion of tungsten has been investigated in stagnant water, flowing water, and irradiation environments [6,7,8]. Depending on temperature, dissolved oxygen, pH, and local solution chemistry, exposed W can form hydrated oxides and other oxygen-containing surface species while releasing soluble W-bearing species into the water. Tantalum cladding has therefore been used to isolate W from coolant in neutron-source targets, including systems developed for CSNS, KENS, and LANSCE [9,10,11]. Its protective behavior is associated with the spontaneous formation of a compact Ta_2_O_5_-rich passive film [12,13,14,15]. Conventional cladding, however, requires demanding forming, joining, or diffusion-bonding procedures and may be constrained by component geometry and interfacial defects.

Magnetron-sputtered Ta films offer an alternative means of introducing a thin corrosion barrier onto W. Compared with bulk cladding, sputtering uses less Ta and provides control over film thickness and microstructure, but the protection depends on coating continuity, density, and interfacial attachment [16]. A continuous nanoscale Ta film is also expected to impose substantially less additional thermal resistance than conventional Ta cladding, thereby better preserving heat transfer from the W substrate. However, this advantage can only be realized when sufficient coating continuity, density, and protective integrity are maintained. Laser ablation provides a non-contact route for tailoring this interface. Nanosecond irradiation produces localized melting, material removal, melt ejection, rapid resolidification, and redeposition, replacing machining grooves with multiscale asperities, depressions, and resolidified features [17,18,19]. These structures can increase the coating/substrate contact area and provide additional nucleation and mechanical-interlocking sites. However, the same laser-generated roughness can increase the exposed area and density of reactive sites when W is left uncoated. Laser ablation should therefore be regarded as an interfacial-engineering treatment rather than an intrinsically corrosion-resistant modification; its effectiveness depends on whether the subsequently deposited coating forms a continuous and adherent barrier. Representative studies and the main distinction of the present thin-film approach are summarized in Table 1.

In the present work, untreated tungsten (W), laser-ablated tungsten (LA-W), and a 500 nm Ta film magnetron-sputtered onto laser-ablated tungsten (Ta-LA-W) were exposed to static deionized water for up to 28 days, representing an approximately one-month shutdown interval. Surface morphology and elemental distribution were examined by SEM/EDS, three-dimensional topography was quantified by laser scanning confocal microscopy (LSCM), phase constitution was assessed by XRD, surface chemical states were analyzed by XPS, and dissolved W and Ta concentrations were measured by ICP-OES. The objectives were to determine how laser ablation modifies the initial W surface and its aqueous response, evaluate the sputtered Ta film as a thin-barrier alternative designed to balance corrosion protection and heat-transfer capability, and clarify the respective roles of surface texturing, W dissolution, and Ta passivation.

## 2. Materials and Methods

### 2.1. Materials

Commercial tungsten plates with a nominal purity of 99.95% and dimensions of 10 × 10 × 2 mm were purchased from Qinghe Haoxuan Metal Material Co., Ltd (Xingtai, China). Before treatment, the specimens were sequentially cleaned in propanol, rinsed with deionized water, and dried overnight at 80 °C. A high-purity Ta target (420 × 80 × 4 mm), purchased from Element Tech Material Co., Ltd. (Weihai, China), was mounted on a Cu backing plate (428 × 88 × 4 mm) to facilitate heat dissipation during magnetron sputtering.

### 2.2. Nanosecond Laser Ablation

Laser surface structuring was performed using a nanosecond pulsed fiber-laser system (K20-CS, Han’s Laser) equipped with an IPG 20 W source, a galvanometric scanner, an EMCC control unit, a motion platform, and the associated workstation software. The wavelength, pulse duration, repetition rate, and nominal spot diameter were 1064 nm, 100 ns, 20 kHz, and 15 μm, respectively. The laser power, scanning speed, hatch spacing, and duty cycle were set to 18 W, 400 mm s^−1^, 15 μm, and 75%, respectively. After processing, the specimens were rinsed with deionized water and dried overnight at 80 °C.

### 2.3. Ta Deposition by Pulsed-DC Magnetron Sputtering

The Ta coating was deposited directly onto the laser-ablated W (LA-W) substrate using a pulsed-DC magnetron sputtering system (JCPF1600, Beijing Technol Science Co., Ltd., Beijing, China). The sputtering chamber was connected to an Ar glove box in which the H2O and O2 concentrations were maintained below 0.01 ppm. The specimens were fixed to the sample holder using a thermally conductive adhesive and transferred into the vacuum chamber, which was evacuated to 8 × 10^−4^ Pa. Deposition was conducted without intentional substrate heating in a cleanroom maintained at 25 °C. The substrate holder was maintained at floating potential, and no external substrate bias was applied. Deposition was performed at 300 W, a duty cycle of 90%, a working pressure of 0.5 Pa, and an Ar flow rate of 20 sccm. The Ta coating thickness was inferred from the deposition duration. In our previous study, the Ta deposition rate under the magnetron sputtering conditions adopted in the present work was experimentally determined to be 12.1 nm/min using Si substrates and verified through coating morphology characterization and step-height measurements using a stylus profilometer [20]. Accordingly, the deposition time was adjusted to obtain a Ta thickness of 500 nm. After sputtering, the chamber was gradually vented to the glove-box atmosphere before the coated specimens were removed.

### 2.4. Static Deionized-Water Immersion

Static immersion was used to represent a shutdown-stage condition in which the target surface remains in contact with stagnant deionized water without beam irradiation. Each specimen was immersed in 25 mL of deionized water with a resistivity of 18.25 MΩ cm for 7, 14, 21, or 28 days. Three separate specimens were tested for each combination of surface condition and immersion duration. Throughout the exposure period, all immersion vessels were placed in a temperature-controlled clean room maintained at 25 °C. After exposure, the specimens were removed, lightly rinsed with ultrapure water, dried at 80 °C, and characterized. The immersion solutions were retained for elemental analysis.

### 2.5. Sample Characterization

Surface morphology and near-surface elemental distribution were examined by field-emission scanning electron microscopy coupled with energy-dispersive X-ray spectroscopy (SEM/EDS, JSM-7800F, Tokyo, Japan). Three-dimensional topography was measured by laser scanning confocal microscopy (LSCM, Leica DCM8, Wetzlar, Germany), and the areal arithmetic mean height, Sa, was obtained from three representative areas on each specimen. Phase analysis was performed by X-ray diffraction (XRD, D8 ADVANCE, Bruker, MA, USA) using Cu Kα radiation over 2θ = 30–80°. Surface chemical states were analyzed by X-ray photoelectron spectroscopy (XPS, ULVAC-PHI GENESIS 500, Kanagawa, Japan) using monochromatic Al Kα radiation with a photon energy of 1486.6 eV. Binding energies were referenced to the C–C/C–H component of adventitious carbon at 284.8 eV [21]. The applicability of this reference to the present conductive W/Ta system was supported by our previous Fermi-edge-referenced measurements on closely related Ta-containing metallic specimens, which yielded consistent chemical-state positions and assignments [20]. The present XPS analysis primarily focuses on relative changes in the fitted chemical-state components under consistent acquisition and fitting conditions, with the assignments additionally evaluated from the characteristic spin–orbit separations and component line shapes. Dissolved W and Ta were measured by inductively coupled plasma optical emission spectroscopy (ICP-OES, Agilent 5110, Wetzlar, Germany) using an RF power of 1250 W, plasma and auxiliary gas flows of 12.0 and 1.0 L min^−1^, respectively, and a sample-uptake delay of 20 s. Each immersion solution was measured in triplicate by ICP-OES as technical repeats.

## 3. Results and Discussion

### 3.1. Initial Surface Morphology and Topography

**Figure 1:**
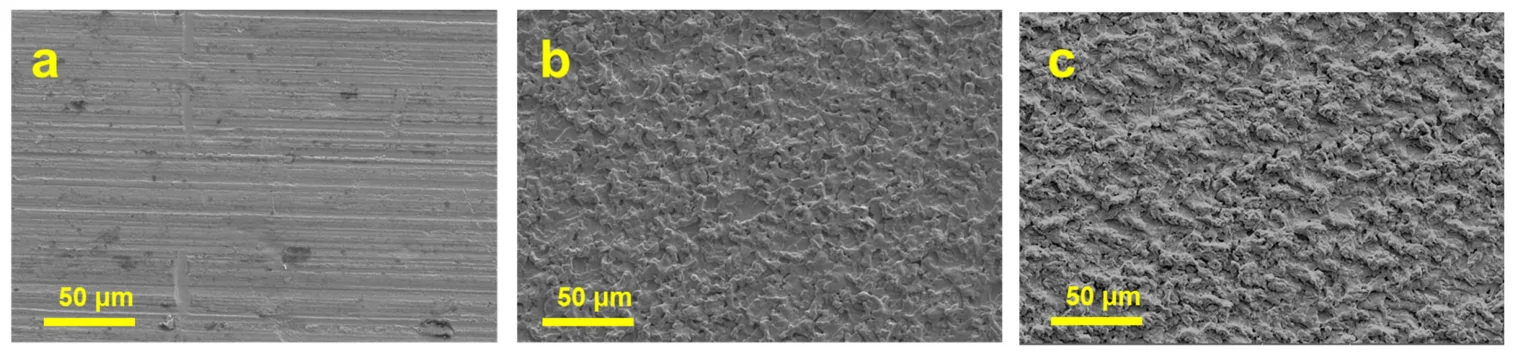
SEM morphologies before immersion: (**a**) W, (**b**) LA-W, and (**c**) Ta-LA-W.

The initial SEM morphologies of W, LA-W, and Ta-LA-W are compared in Figure 1. Pristine W exhibits parallel machining or grinding grooves with sparse particulate features (Figure 1a), and the surface is dominated by a long-range directional texture. Laser ablation removes most of this directional character and produces an interconnected morphology consisting of irregular ridges, shallow depressions, resolidified protrusions, and finer secondary features (Figure 1b). These structures originate from localized melting, material removal, melt ejection, rapid solidification, and redeposition during nanosecond laser–material interaction. After magnetron sputtering, Ta-LA-W retains the laser-generated relief while developing a finer granular and locally flake-like morphology (Figure 1c). The 500 nm Ta coating follows the underlying peaks and valleys rather than planarizing the substrate and partially fills shallow depressions. Preservation of the multiscale topography increases the geometric coating/substrate contact area and may promote mechanical interlocking, as reported for coatings deposited on laser-structured substrates [22,23,24].

**Figure 2:**
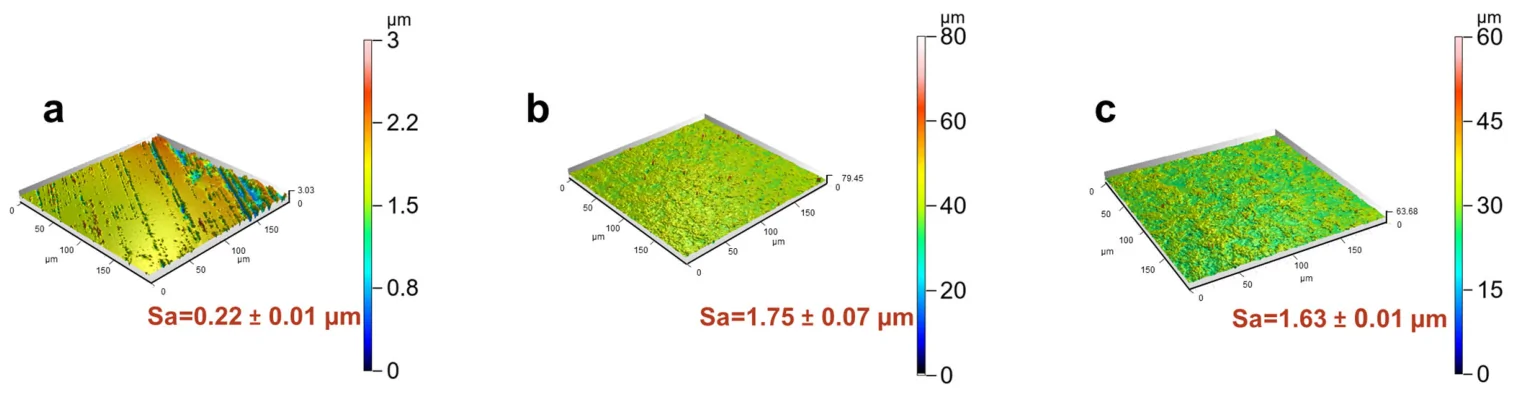
Initial three-dimensional LSCM topographies and corresponding Sa values of (**a**) W, (**b**) LA-W, and (**c**) Ta-LA-W (The Sa values are based on three measurements).

The LSCM topographies quantify the morphological transformation (Figure 2). Pristine W has Sa = 0.22 ± 0.01 μm, whereas laser ablation increases Sa to 1.75 ± 0.07 μm. After Ta deposition, Sa decreases slightly to 1.63 ± 0.01 μm, consistent with partial coverage of sharp asperities and shallow filling of valleys while most of the laser-induced relief is retained. The similar roughness values of LA-W and Ta-LA-W indicate that the coating architecture remains governed by the prestructured substrate. Such substrate features can increase the density of nucleation sites, shorten the uninterrupted lateral migration distance of arriving species, and influence the coalescence of sputtered Ta [25,26].

### 3.2. Surface Morphology and Elemental Distribution During Immersion

**Figure 3:**
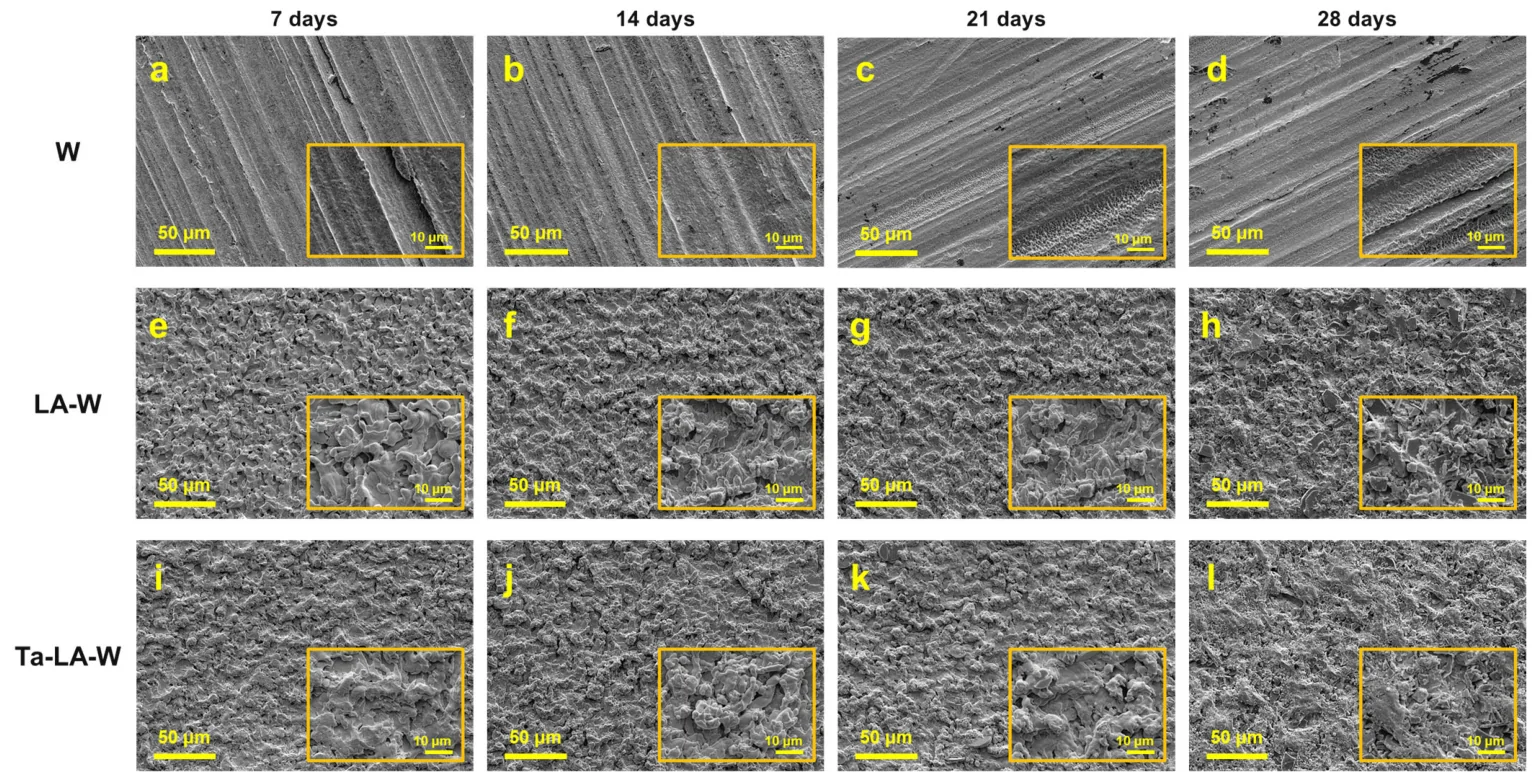
SEM morphologies after static deionized-water immersion for 7, 14, 21, and 28 days: (**a**–**d**) W, (**e**–**h**) LA-W, and (**i**–**l**) Ta-LA-W, respectively. The orange-bordered insets provide higher-magnification views of representative local regions.

Figure 3 compares the SEM morphologies after 7, 14, 21, and 28 days of static deionized-water immersion. For W (Figure 3a–d), the original parallel grooves remain visible throughout the test, while their contrast and the population of fine surface features change with time. Local bright and dark regions in the higher-magnification views indicate nonuniform formation, redistribution, or removal of surface species. LA-W follows a different evolution (Figure 3e–h). The laser-generated network provides a larger exposed area and a higher density of edges, resolidified regions, and local defects than pristine W. During immersion, the surface becomes progressively more heterogeneous, and coarser plate-like or agglomerated features develop at intermediate times. Preferential reaction at high-curvature or defect-rich sites, local dissolution of fine asperities, and redistribution of W-containing products can contribute to this morphology; increased laser-generated area has likewise been associated with changes in aqueous degradation of textured metallic surfaces [27]. Ta-LA-W retains a comparatively uniform textured morphology at all four durations (Figure 3i–l), without an evident large-area delamination or connected through-coating crack network in the examined fields.

**Figure 4:**
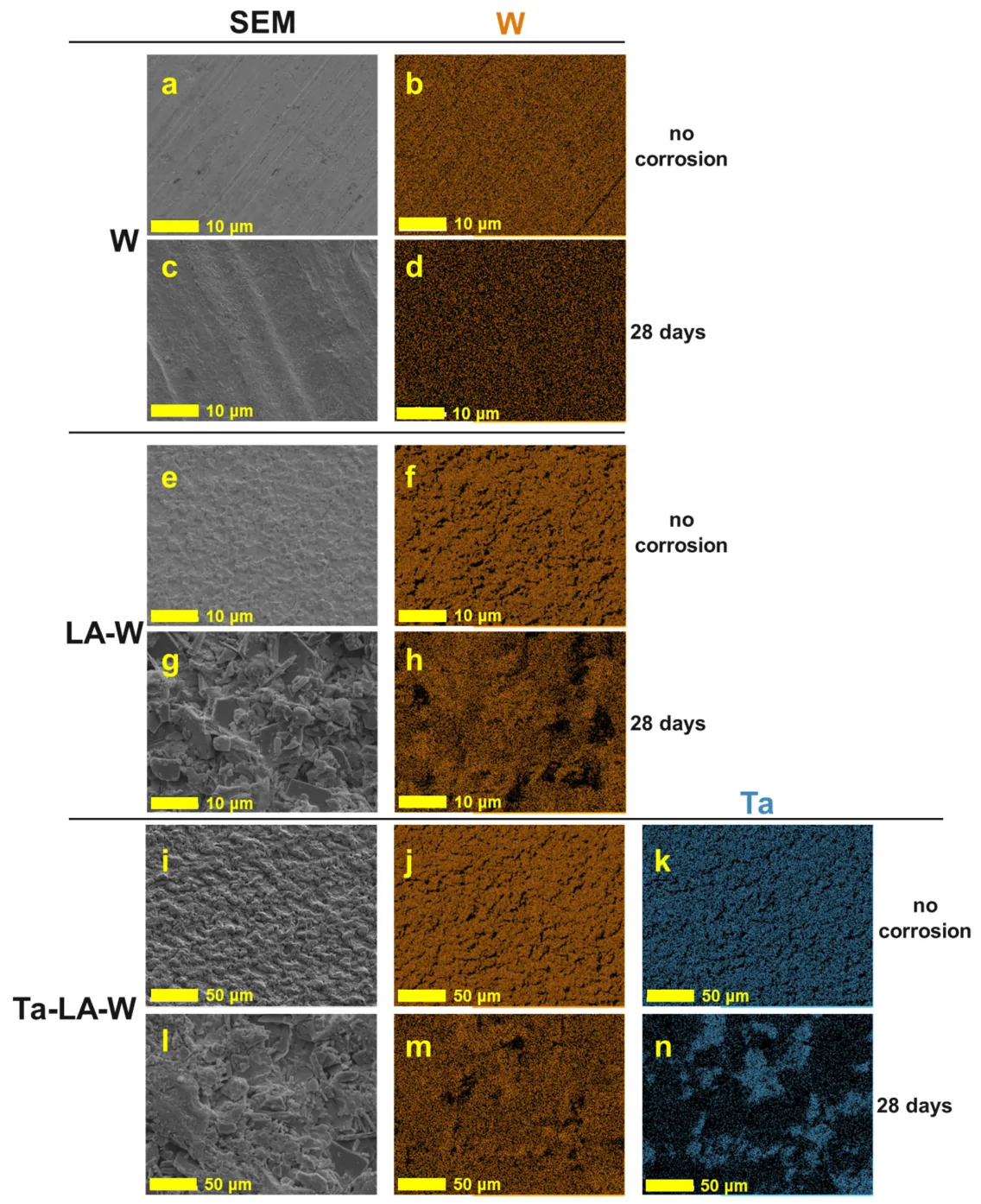
SEM images and EDS elemental maps before immersion and after 28 days. W: (**a**) SEM and (**b**) W map before immersion; (**c**) SEM and (**d**) W map after 28 days. LA-W: (**e**) SEM and (**f**) W map before immersion; (**g**) SEM and (**h**) W map after 28 days. Ta-LA-W: (**i**) SEM, (**j**) W map, and (**k**) Ta map before immersion; (**l**) SEM, (**m**) W map, and (**n**) Ta map after 28 days.

The SEM/EDS comparison in Figure 4 shows the elemental distributions before immersion and after 28 days. For W, the W signal remains continuous across the observed area, and local intensity variations mainly follow the surface topography and SEM contrast. The W distribution of LA-W is more heterogeneous because of the pronounced laser-generated relief. After immersion, the surface remains W-rich, although the map becomes locally less uniform as the topography changes and corrosion products are distributed nonuniformly. For Ta-LA-W, both W and Ta are detected before immersion, and Ta remains distributed across the mapped field after 28 days. The patchier post-immersion Ta map may arise from local variations in surface inclination, coating thickness, or oxide-film thickness. The field-wide Ta signal and the absence of obvious large-area spallation indicate substantial retention of the Ta-containing layer, while localized thinning cannot be excluded from EDS mapping alone. These static-immersion results provide a basis for selecting the Ta coating parameters in subsequent irradiation studies, in which coating integrity will be further evaluated.

### 3.3. Three-Dimensional Topographic Evolution

**Figure 5:**
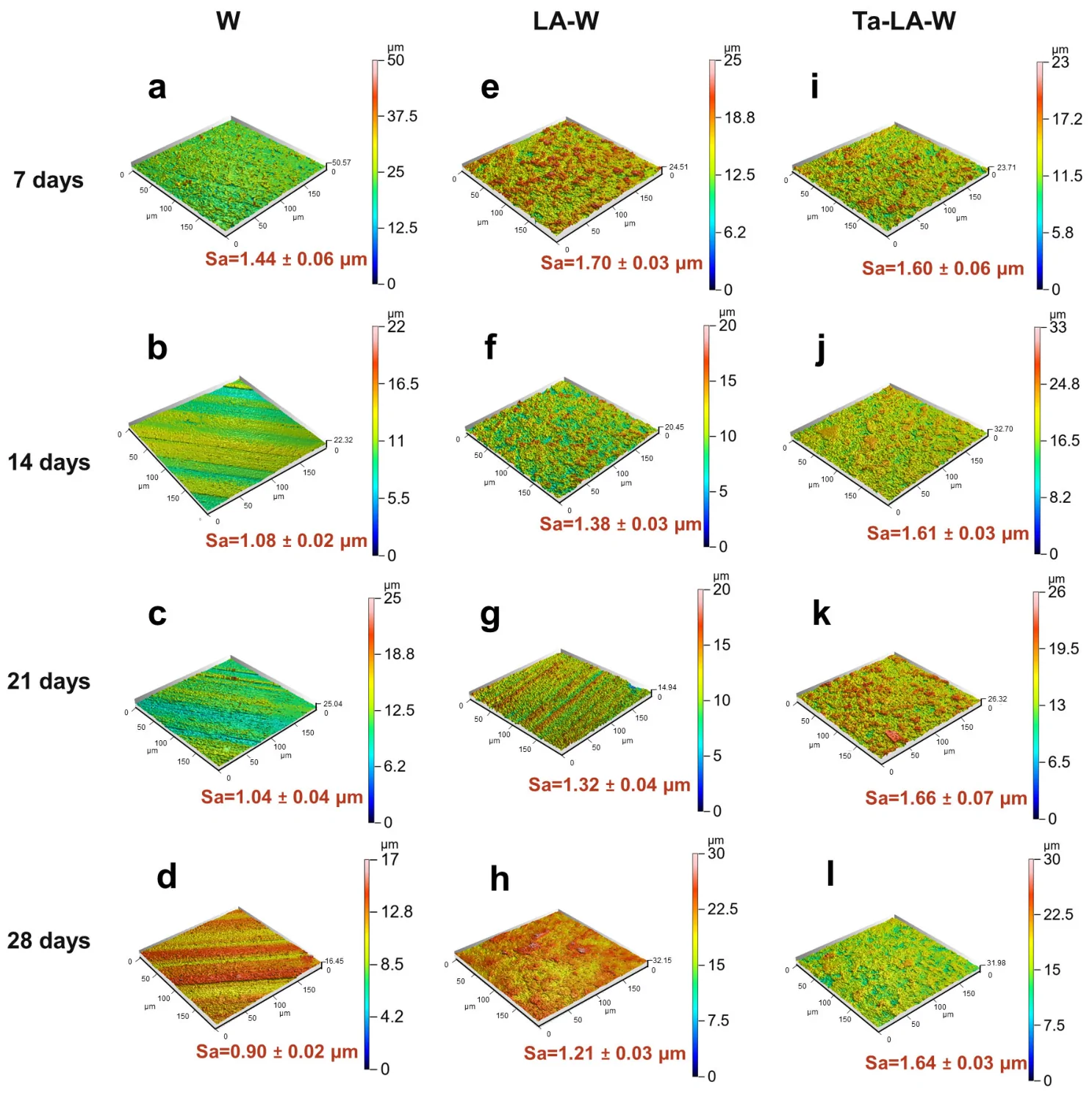
Three-dimensional LSCM topographies and corresponding roughness after static deionized-water immersion for 7, 14, 21, and 28 days: (**a**–**d**) W, (**e**–**h**) LA-W, and (**i**–**l**) Ta-LA-W, respectively (The Sa values are based on three measurements).

Figure 5 summarizes the topographic evolution during immersion. For W, Sa is 1.44 ± 0.06, 1.08 ± 0.02, 1.04 ± 0.04, and 0.90 ± 0.02 μm after 7, 14, 21, and 28 days, respectively, compared with 0.22 ± 0.01 μm before immersion. The early increase reflects the development of additional height contrast on the initially machined surface, which may arise from localized attack and nonuniform product accumulation. The subsequent decrease can result from dissolution or detachment of protruding products, preferential removal of elevated regions, and redistribution or reprecipitation of dissolved species. For LA-W, Sa decreases monotonically from 1.75 ± 0.07 μm to 1.70 ± 0.03, 1.38 ± 0.03, 1.32 ± 0.04, and 1.22 ± 0.03 μm. The progressive attenuation is attributed to preferential reaction of high-curvature asperities and resolidified features, together with partial filling or coverage of valleys. The laser-generated surface therefore loses approximately one-third of its initial roughness during 28 days of immersion. Ta-LA-W behaves differently: Sa remains between 1.60 ± 0.06 and 1.66 ± 0.07 μm, compared with 1.63 ± 0.01 μm initially. The limited 0.06 μm range indicates that the textured topography was largely retained beneath the Ta coating.

### 3.4. Phase Constitution Before and After Immersion

**Figure 6:**
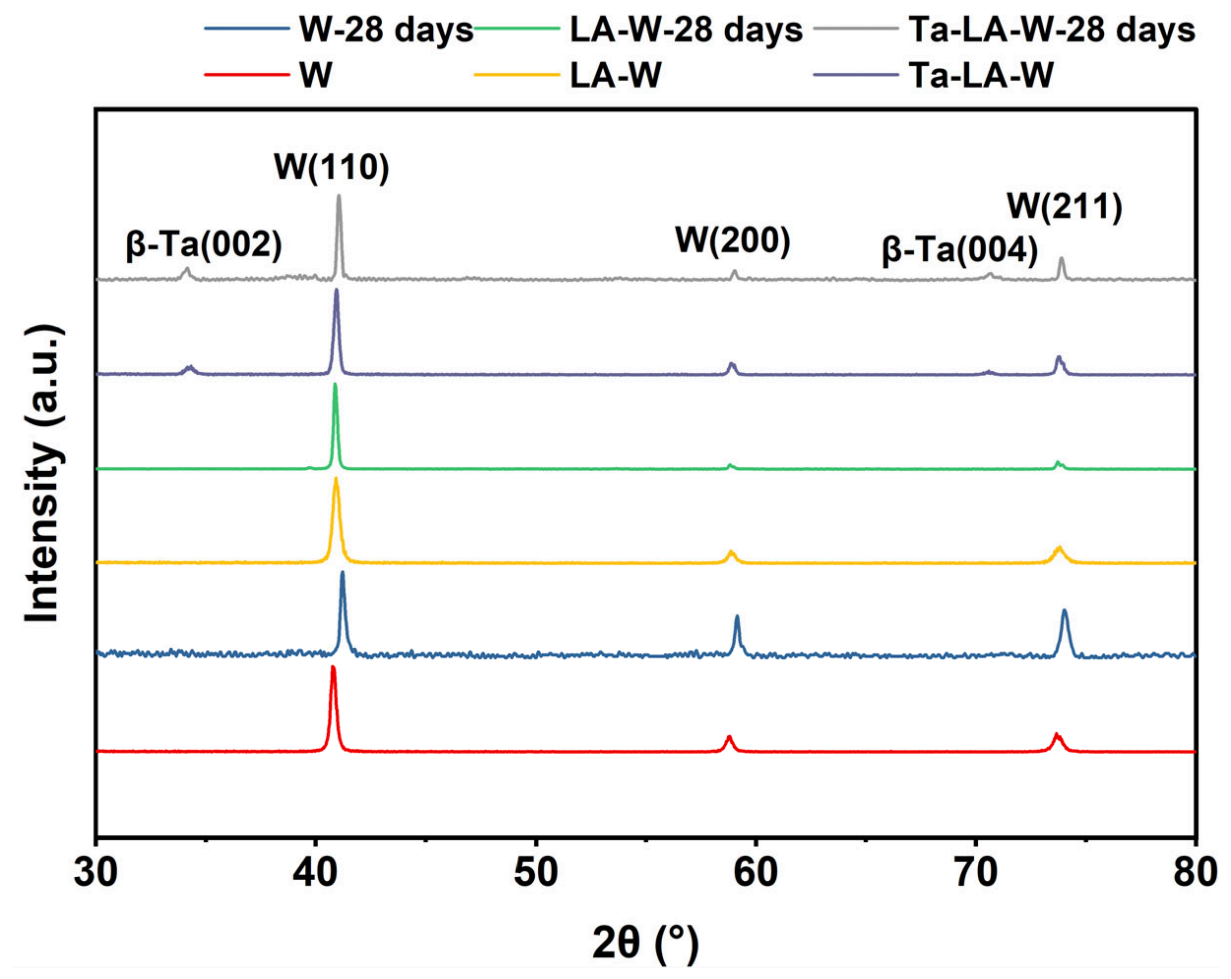
XRD patterns of W, LA-W, and Ta-LA-W before immersion and after 28 days. Patterns are normalized and vertically offset; displayed intensities are not used for quantitative phase comparison.

The XRD patterns of W, LA-W, and Ta-LA-W are shown in Figure 6. All specimens exhibit peaks at 2θ = 41.04°, 59.02°, and 73.88°, assigned to the (110), (200), and (211) planes of body-centered-cubic α-W, respectively. A slight shift in the W(110) reflection is observed after 28 days of immersion, which may result from near-surface strain and residual-stress redistribution associated with oxidation and dissolution; minor effects from surface roughness and specimen positioning cannot be excluded. Nevertheless, the other α-W reflections remain essentially unchanged, indicating no detectable transformation of the bulk W phase. Their positions remain essentially unchanged after laser ablation and after 28 days of immersion, indicating that the treatments affected the near-surface region without producing a detectable change in the bulk W phase. Ta-LA-W additionally exhibits reflections at approximately 34.16° and 70.68°, indexed to β-Ta(002) and β-Ta(004), respectively. β-Ta is commonly obtained in sputter-deposited Ta films, and its formation depends on deposition pressure, texture development, impurity incorporation, and growth stress [28,29,30,31]. The β-Ta reflections remain visible after immersion. No distinct crystalline tungsten-oxide or tantalum-oxide peaks are resolved, which is consistent with oxide layers that are thin, poorly crystalline, or amorphous.

### 3.5. Surface Chemical States

**Figure 7:**
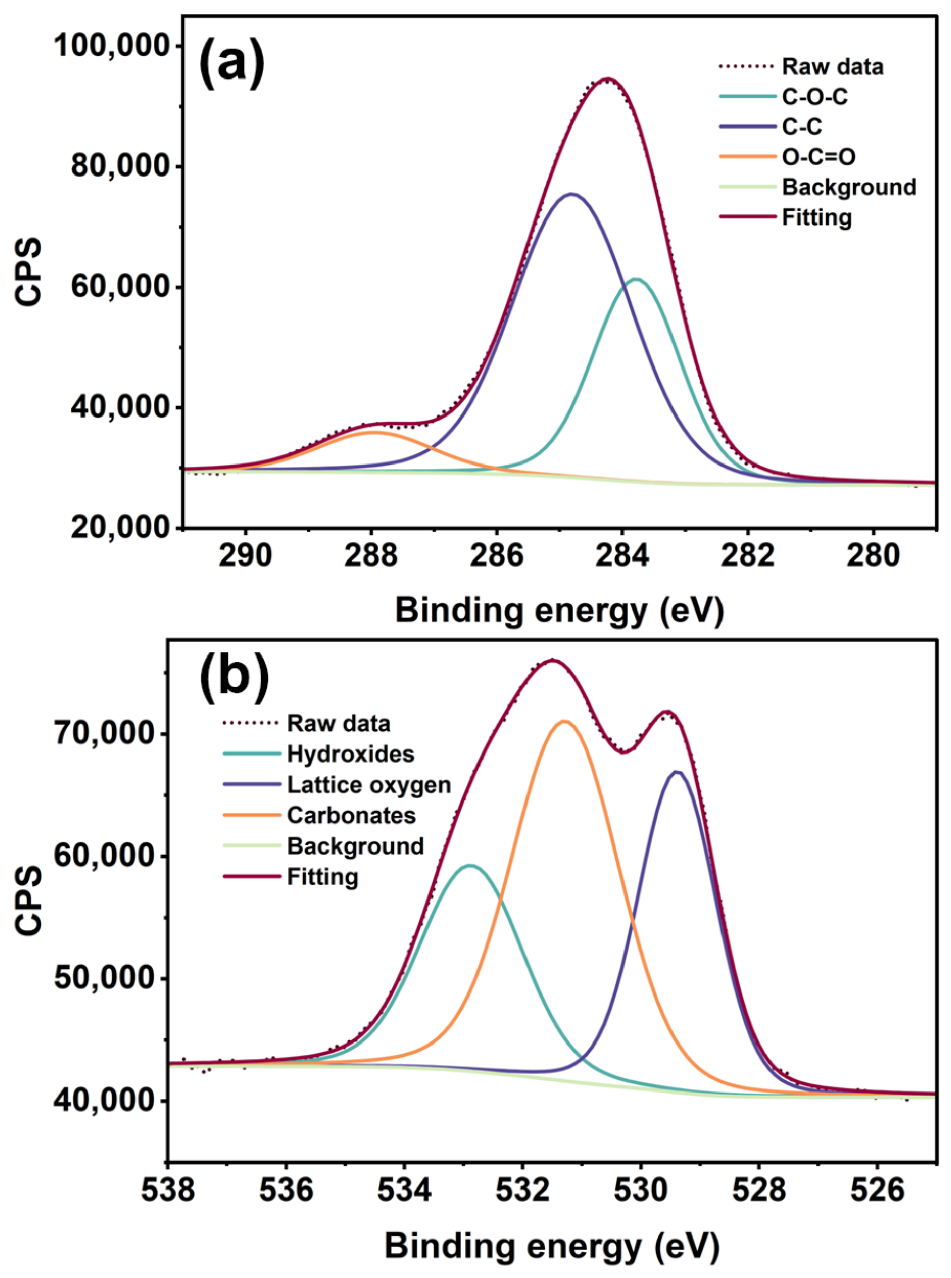
Representative high-resolution XPS spectra acquired from initial W: (**a**) C 1s and (**b**) O 1s.

Figure 7 presents representative high-resolution C 1s and O 1s spectra acquired from W. The C 1s envelope was fitted with components assigned to C–C, C–O–C, and O–C=O at approximately 284.8, 283.8, and 288.0 eV, respectively (Figure 7a). The O 1s envelope contains contributions from lattice oxygen in metal oxides near 529.4–530.0 eV, oxygen-containing or carbonate species near 531.3 eV, and hydroxyl groups or molecularly adsorbed water near 532.9 eV (Figure 7b). Comparable C- and O-containing contributions were present on the other specimen states; W is therefore shown as a representative example. The detailed XPS peak-fitting parameters and relative peak areas are provided in Table S1.

**Figure 8:**
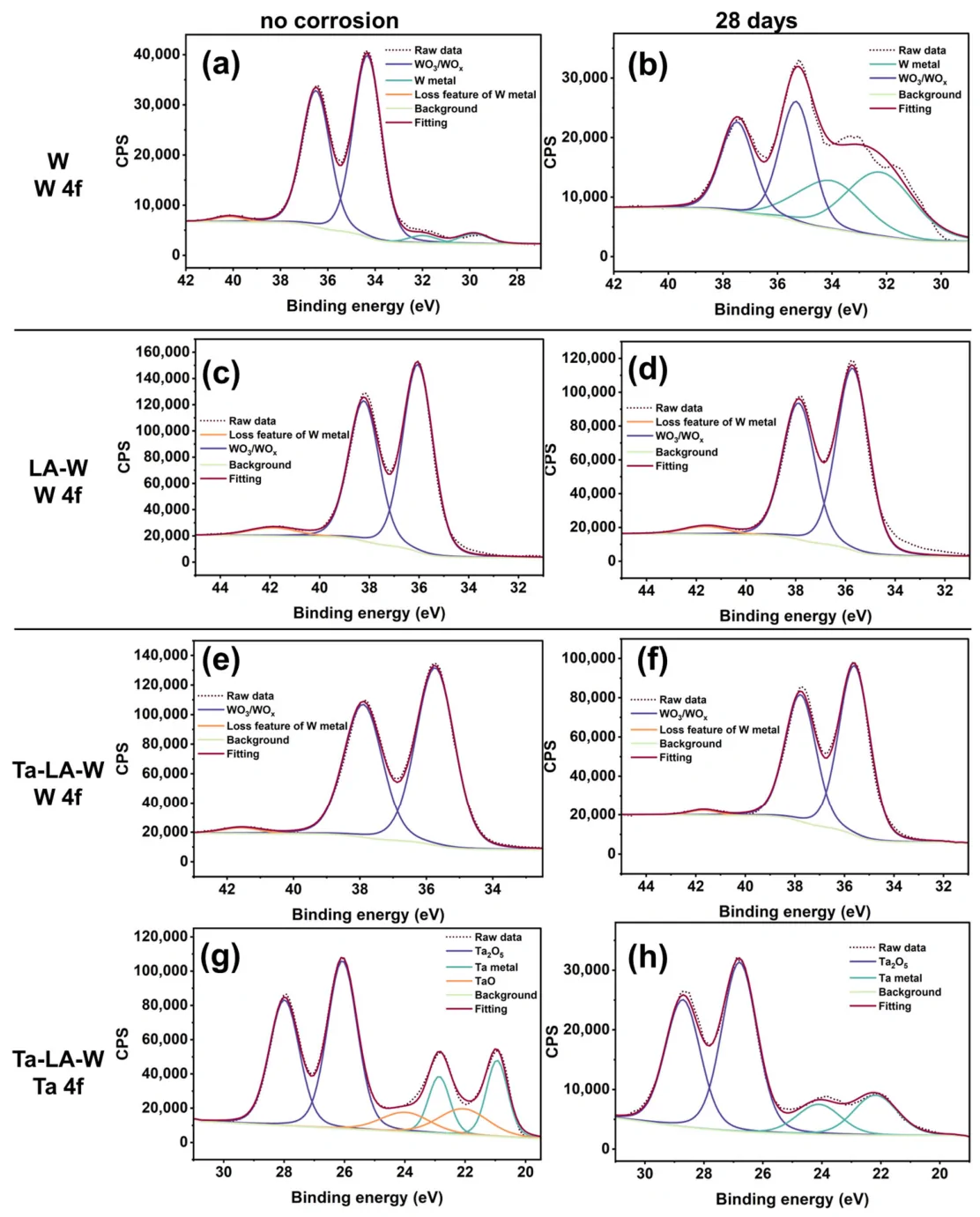
High-resolution XPS spectra before and after 28 days of immersion: W 4f spectra of (**a**,**b**) W, (**c**,**d**) LA-W, and (**e**,**f**) Ta-LA-W before and after immersion, respectively; Ta 4f spectra of Ta-LA-W (**g**) before and (**h**) after immersion.

The W 4f spectra of W, LA-W, and Ta-LA-W are compared in Figure 8a–f. Metallic W is represented by the W 4f7/2 and W 4f5/2 components at approximately 29.8 and 32.0 eV, whereas WOx-related components occur near 34.3 and 36.5 eV; the broad feature around 40–42 eV is assigned to the energy-loss structure associated with metallic W [32]. For untreated W, the WOx-related contribution increases after 28 days and the metallic-W loss feature disappears (Figure 8a,b), indicating accumulation of an oxide-rich surface layer. Conversion of the exposed surface from metallic W toward WOx is undesirable for a target–coolant interface because it changes the intended metallic surface state and can contribute to surface-product accumulation during service. LA-W retains a broadly similar W 4f structure before and after immersion (Figure 8c,d). In conjunction with its high dissolved W concentration, the absence of pronounced WOx enrichment indicates sustained dissolution and removal of W-bearing products rather than their retention as a stable oxide-rich layer. Ta-LA-W also retains a similar W 4f envelope (Figure 8e,f), but here the low W release identifies protection by the Ta-containing barrier as the principal reason for the limited W chemical-state change. Before immersion, the Ta 4f spectrum was fitted with three spin–orbit doublets assigned to metallic Ta, sub-stoichiometric TaO, and Ta_2_O_5_ (Figure 8g).

The metallic-Ta 4f_7_/_2_ component is located at approximately 20.9 eV, consistent with a previously reported value for metallic Ta [33]. With the Ta 4f spin–orbit separation constrained to 1.92 eV, the corresponding Ta 4f_5_/_2_ component occurs at approximately 22.9 eV after rounding. The Ta_2_O_5_ components occur at approximately 26.1 and 28.0 eV [34,35]. The identification of an intermediate TaO component is consistent with detailed analyses of Ta-based films showing mixed suboxide species at the transition between Ta_2_O_5_ and metallic Ta [36]. In the present spectrum, TaO accounts for 16.12% of the total fitted Ta 4f peak area. After 28 days of immersion, the TaO component is no longer resolved, whereas metallic Ta and Ta_2_O_5_ remain detectable (Figure 8h). The disappearance of TaO is consistent with further oxidation of the lower-valence Ta suboxide during immersion. The remaining Ta_2_O_5_/metallic-Ta structure corresponds to a Ta_2_O_5_-rich passive surface over metallic Ta, a configuration associated with the aqueous passivity of tantalum [37].

### 3.6. Dissolved W and Ta Concentrations

**Figure 9:**
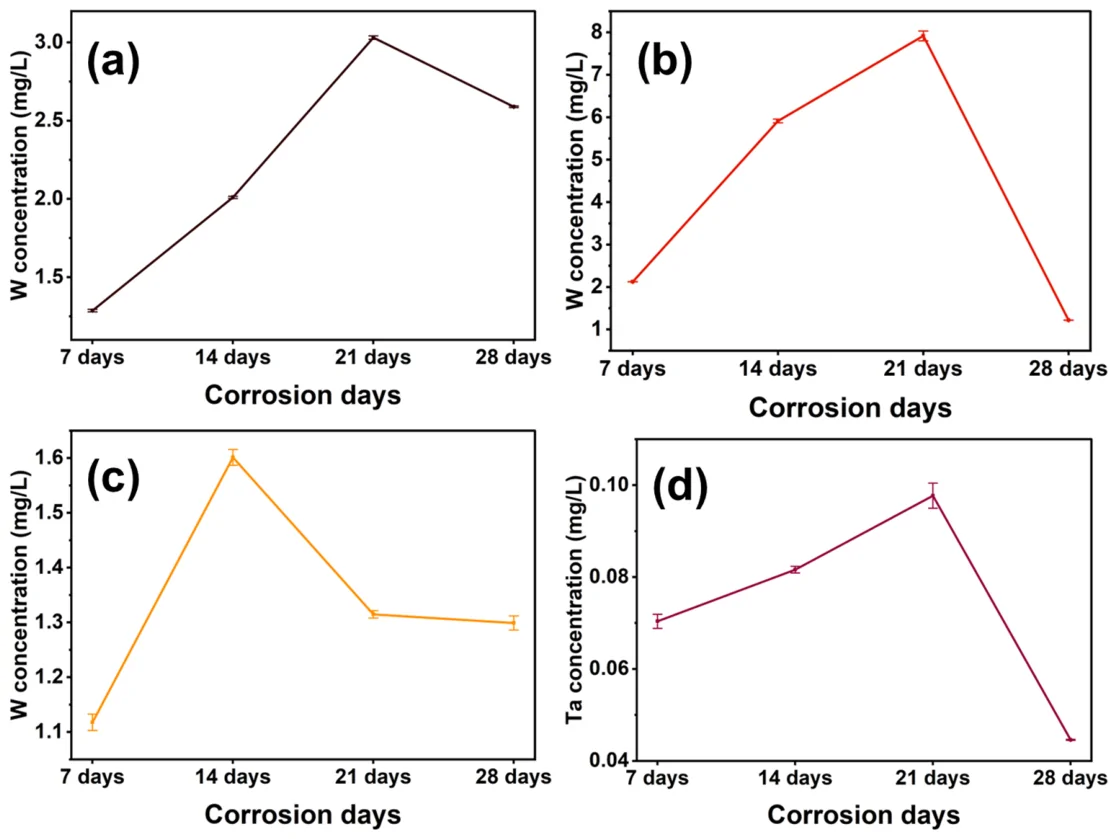
Dissolved-metal concentrations during static immersion: W concentrations in solutions exposed to (**a**) W, (**b**) LA-W, and (**c**) Ta-LA-W; (**d**) Ta concentration in solutions exposed to Ta-LA-W (The concentrations are based on three measurements).

ICP-OES was used to quantify W and Ta in the immersion solutions (Figure 9). For untreated W, the dissolved W concentration increases from 1.286 mg·L^−1^ at 7 days to 2.010 and 3.030 mg·L^−1^ at 14 and 21 days, respectively, before decreasing to 2.588 mg·L^−1^ at 28 days. The increase during the first 21 days reflects continued interaction between the exposed W surface and deionized water. Together with the post-immersion W 4f change, the solution data indicate concurrent surface oxidation and W release; the oxide-containing layer does not completely suppress transport of W-bearing species into the water.

LA-W shows substantially higher dissolved W concentrations during the first 21 days, reaching 2.122, 5.913, and 7.915 mg·L^−1^ after 7, 14, and 21 days, respectively. Laser ablation produces ridges, depressions, resolidified protrusions, and redeposited features that increase the real contact area between W and water. High-curvature and rapidly resolidified regions also provide additional sites for interfacial reaction, and surface engineering has been shown to alter the aqueous degradation of W-based materials [38]. Consequently, LA-W releases more W than untreated W. Its W 4f structure nevertheless remains broadly unchanged, indicating that the generated W-bearing species are dissolved, detached, or redistributed rather than retained as a stable oxide-rich surface layer. The solution concentration decreases to 1.218 mg·L^−1^ at 28 days. Ta-LA-W maintains lower W concentrations of 1.118, 1.601, 1.315, and 1.299 mg·L^−1^ after 7, 14, 21, and 28 days, respectively. At 21 days, this value is 83.4% lower than that of LA-W and 56.6% lower than that of W. Dissolved Ta remains below 0.10 mg·L^−1^, with concentrations of 0.0704, 0.0816, 0.0977, and 0.0446 mg·L^−1^. The low W and Ta concentrations are attributed to the 500 nm Ta barrier and its Ta_2_O_5_-containing passive surface. Because ICP-OES measures the elemental concentration remaining in each recovered solution rather than cumulative mass loss, the decreases at 28 days may reflect precipitation, adsorption on the specimen or vessel, or incorporation into surface products.

### 3.7. Corrosion and Protection Mechanism

**Figure 10:**
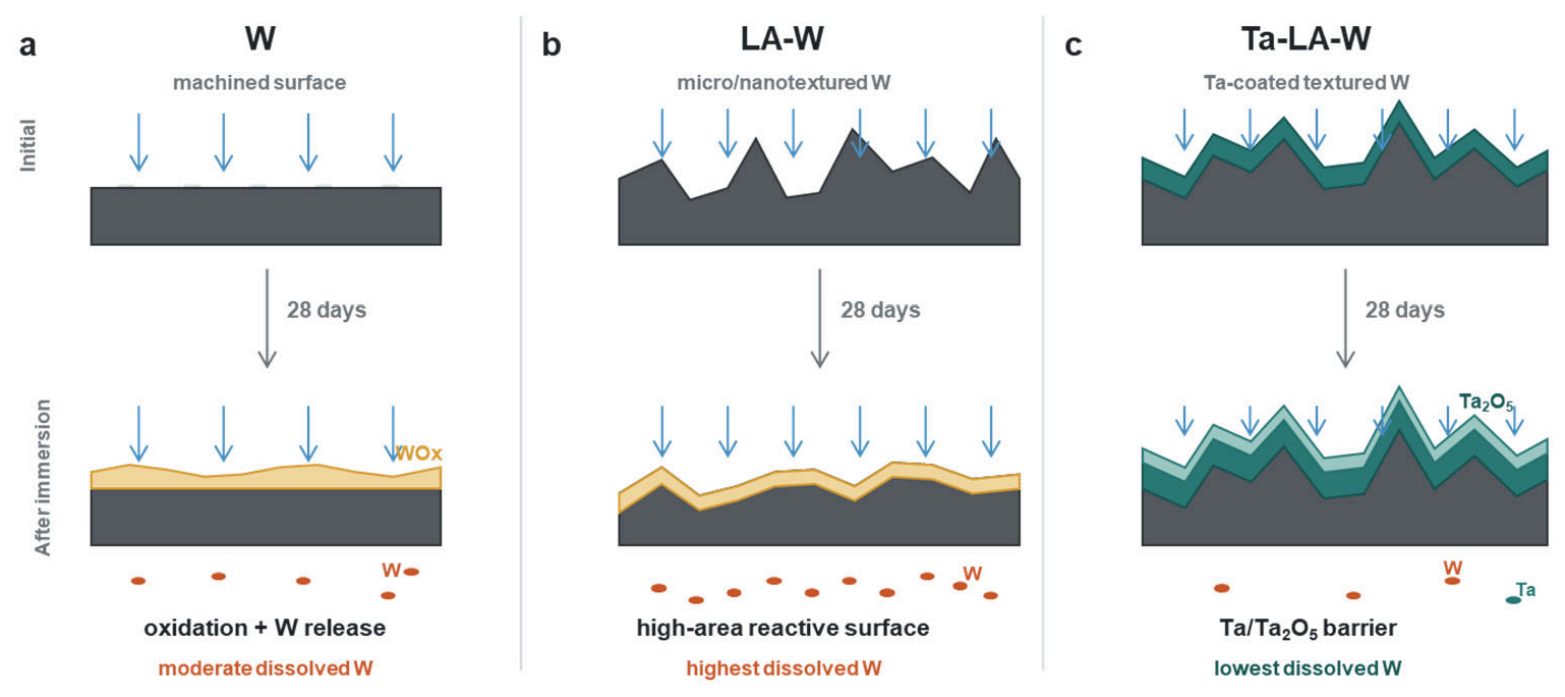
Proposed corrosion and protection mechanisms of (**a**) W, (**b**) LA-W, and (**c**) Ta-LA-W during static deionized-water immersion.

Figure 10 summarizes the corrosion responses of the three surfaces. Untreated W undergoes concurrent oxidation and dissolution: WOx accumulates on the exposed surface, while W-bearing species continue to enter the water. The resulting oxide-rich surface differs from the metallic W state required for the target interface. For LA-W, laser-generated ridges, valleys, and resolidified features increase the effective contact area and the number of reactive sites. The corresponding topographic attenuation and high dissolved W concentrations indicate sustained dissolution, while the similar W 4f envelopes before and after immersion indicate limited retention of a stable oxide-rich layer. For Ta-LA-W, the 500 nm Ta coating follows the laser-textured substrate and carries a Ta_2_O_5_-containing passive surface. This Ta/Ta_2_O_5_ barrier limits direct water access to W, accounting for the stable Sa, limited change in the W 4f envelope, and low dissolved W concentration. Under the present beam-off condition, the dissolution-based sequence is therefore LA-W > W > Ta-LA-W. Laser texturing alone accelerates the corrosion of bare W, whereas the same architecture becomes beneficial when used as the interface for a retained Ta coating.

## 4. Conclusions

A laser-structured W/Ta surface architecture was examined under 28 days of static deionized-water exposure. The principal conclusions are as follows. (1) Laser ablation converted the machined W surface (Sa = 0.22 ± 0.01 μm) into a hierarchical micro/nanostructure (Sa = 1.75 ± 0.07 μm), and the 500 nm Ta coating retained most of this architecture (Sa = 1.63 ± 0.01 μm). (2) Laser ablation alone increased aqueous degradation: LA-W showed progressive topographic attenuation and reached 7.915 mg·L^−1^ dissolved W at 21 days, compared with 3.030 mg·L^−1^ for untreated W. Its unchanged W 4f envelope is attributed to sustained dissolution and limited retention of W oxide products. (3) Untreated W developed a stronger WOx-related surface contribution and lost the metallic-W loss feature after immersion, indicating conversion of the exposed metallic surface toward an oxide-rich state. (4) Ta-LA-W maintained Sa within the range from 1.60 ± 0.06 to 1.66 ± 0.07 μm and limited dissolved W to 1.118–1.601 mg·L^−1^. The retained β-Ta reflections and Ta_2_O_5_/metallic-Ta components indicate a Ta barrier with a passive oxide-covered surface. (5) Collectively, these results clarify the dual role of laser structuring in the aqueous degradation of W. When W is left uncoated, the increased exposed area and density of reactive sites accelerate dissolution; when followed by the deposition of a continuous Ta film, the laser-generated structure serves as an interfacial-engineering platform for the protective barrier. Therefore, surface roughening alone does not provide corrosion resistance, and the continuity and passivation of the subsequently deposited Ta film are the determining factors. The 500 nm Ta-film architecture provides a potential route to suppress W release during shutdown while avoiding the thickness of conventional Ta cladding and minimizing the associated additional thermal resistance. These findings provide a basis for selecting Ta-film thickness and deposition parameters for water-cooled W targets. The present study establishes a static, beam-off corrosion baseline and does not reproduce radiolysis, irradiation damage, high heat flux, thermal cycling, or flow-assisted corrosion during operation. Future work will combine the present results with irradiation experiments and further evaluate localized coating thinning, defect development, and long-term interfacial integrity under coupled operating conditions.

## Figures and Tables

**Table 1:** Comparison of representative studies on the aqueous corrosion and protection of W targets.

Material	Corrosion Environment	Main Outcome	Reference
Bulk Ta-clad W target	Water under different temperatures and flow velocities	Engineering-scale Ta cladding was used to isolate W from the cooling water.	[3]
Irradiated W	Flowing deionized water under proton irradiation	Irradiation, water corrosion, and flow jointly contributed to W loss.	[6]
Uncoated W	Static ultrapure and tap water	Time-dependent dissolution and surface oxidation of W were demonstrated.	[8]
Thin Ta film on laser ablated W	Static deionized water	The nanoscale Ta film substantially reduced W release while minimizing the additional thermal resistance associated with conventional bulk Ta cladding.	Present work

## Data Availability

The data presented in this study are available on request from the corresponding author.

## Supplementary Materials

The following supporting information can be downloaded at: https://www.mdpi.com/article/10.3390/nano16161043/s1, Table S1: XPS peak-fitting parameters and relative peak areas of the specimens of Figure 7 and Figure 8.
