# Supplementary material for: Laser-Ablated Tungsten as an Interfacial Architecture for Tantalum Coatings: Suppression of Shutdown-Stage Water Corrosion in Accelerator-Driven Neutron-Source Targets
Source: Nanomaterials (Basel). 2026 Aug 21;16(16):1043. doi: 10.3390/nano16161043 (PMC13516963; doi:10.3390/nano16161043)
Supplement: Supplementary file 1 [file nanomaterials-16-01043-s001.zip › nanomaterials-4476903-supplementary.pdf]

Table S1. XPS peak-fitting parameters and relative peak areas of the specimens of Figure 7 and Figure 8

Specimen	Spectral region	Peak assignment	Binding energy (eV)	FWHM	Relative peak area (%)	Corresponding figures
W<br>no corrosion	C 1s	C-C	284.8	2.25	60.17	Figure 7a
		C-O-C	283.8	1.65	31.63	
		O-C=O	288.0	2.18	8.19	
	O 1s	Hydroxides	532.9	2.04	24.84	Figure 7b
		Lattice oxygen	529.4	1.56	29.51	
		Carbonates	531.3	2.15	45.65	
	W 4f	WO_3/WO_x	W $4f_{7/2}$: 34.3<br>W $4f_{5/2}$: 36.5	W $4f_{7/2}$:1.41<br>W $4f_{5/2}$: 1.41	W $4f_{7/2}$: 53.20<br>W $4f_{5/2}$: 39.90	Figure 8a
		W metal	W $4f_{7/2}$: 29.8<br>W $4f_{5/2}$: 32.0	W $4f_{7/2}$:1.47<br>W $4f_{5/2}$: 1.47	W $4f_{7/2}$: 3.06<br>W $4f_{5/2}$: 2.29	
		Loss feature of W metal	40.2	1.40	1.54	
W<br>28 days	W 4f	WO_3/WO_x	W $4f_{7/2}$: 35.3<br>W $4f_{5/2}$: 37.5	W $4f_{7/2}$:1.42<br>W $4f_{5/2}$: 1.42	W $4f_{7/2}$: 27.23<br>W $4f_{5/2}$: 20.42	Figure 8b
		W metal	W $4f_{7/2}$: 32.2<br>W $4f_{5/2}$: 34.0	W $4f_{7/2}$: 2.91<br>W $4f_{5/2}$: 2.91	W $4f_{7/2}$: 29.91<br>W $4f_{5/2}$: 22.43	
LA-W<br>no corrosion	W 4f	WO_3/WO_x	W $4f_{7/2}$: 36.0<br>W $4f_{5/2}$: 38.2	W $4f_{7/2}$: 1.43<br>W $4f_{5/2}$: 1.43	W $4f_{7/2}$: 54.91<br>W $4f_{5/2}$: 41.18	Figure 8c
		Loss feature of W metal	41.9	2.33	3.92	
LA-W<br>28 days	W 4f	WO_3/WO_x	W $4f_{7/2}$: 35.7<br>W $4f_{5/2}$: 37.9	W $4f_{7/2}$: 1.53<br>W $4f_{5/2}$: 1.53	W $4f_{7/2}$: 55.28<br>W $4f_{5/2}$: 41.46	Figure 8d
		Loss feature of W metal	41.6	2.08	3.26	
Ta-LA-W<br>no corrosion	W 4f	WO_3/WO_x	W $4f_{7/2}$: 35.7<br>W $4f_{5/2}$: 37.9	W $4f_{7/2}$: 1.37<br>W $4f_{5/2}$: 1.37	W $4f_{7/2}$: 56.09<br>W $4f_{5/2}$: 42.07	Figure 8e
		Loss feature of W metal	41.6	1.38	1.84	

	Ta 4f	Ta_2O_5	Ta $4f_{7/2}$: 26.1<br>Ta $4f_{5/2}$: 28.0	Ta $4f_{7/2}$: 1.17<br>Ta $4f_{5/2}$: 1.17	Ta $4f_{7/2}$: 36.23<br>Ta $4f_{5/2}$: 27.17	Figure 8g
		TaO	Ta $4f_{7/2}$: 22.1<br>Ta $4f_{5/2}$: 24.0	Ta $4f_{7/2}$: 1.20<br>Ta $4f_{5/2}$: 1.20	Ta $4f_{7/2}$: 9.21<br>Ta $4f_{5/2}$: 6.91	
		Ta metal	Ta $4f_{7/2}$: 20.9<br>Ta $4f_{5/2}$: 22.9	Ta $4f_{7/2}$: 0.83<br>Ta $4f_{5/2}$: 0.83	Ta $4f_{7/2}$: 11.70<br>Ta $4f_{5/2}$: 8.78	
Ta-LA-W 28 days	W 4f	WO_3/WO_x	W $4f_{7/2}$: 35.6<br>W $4f_{5/2}$: 37.8	W $4f_{7/2}$: 1.48<br>W $4f_{5/2}$: 1.48	W $4f_{7/2}$: 56.28<br>W $4f_{5/2}$: 42.20	Figure 8f
		Loss feature of W metal	41.7	1.33	1.52	
	Ta 4f	Ta_2O_5	Ta $4f_{7/2}$: 26.8<br>Ta $4f_{5/2}$: 28.7	Ta $4f_{7/2}$: 1.40<br>Ta $4f_{5/2}$: 1.40	Ta $4f_{7/2}$: 44.16<br>Ta $4f_{5/2}$: 33.12	Figure 8h
		Ta metal	Ta $4f_{7/2}$: 22.2<br>Ta $4f_{5/2}$: 24.1	Ta $4f_{7/2}$: 1.74<br>Ta $4f_{5/2}$: 1.74	Ta $4f_{7/2}$: 12.98<br>Ta $4f_{5/2}$: 9.73	
